# RNA-Sequencing Analysis of Gene-Expression Profiles in the Dorsal Gland of *Alligator sinensis* at Different Time Points of Embryonic and Neonatal Development

**DOI:** 10.3390/life12111787

**Published:** 2022-11-04

**Authors:** Haitao Nie, Yuqian Zhang, Shulong Duan, Ying Zhang, Yunlu Xu, Jixiang Zhan, Yue Wen, Xiaobing Wu

**Affiliations:** Anhui Provincial Key Laboratory of the Conservation and Exploitation of Biological Resources, College of Life Sciences, Anhui Normal University, Wuhu 241000, China

**Keywords:** *Alligator sinesis*, dorsal gland, high-throughput RNA sequencing, holocrine secretion, skin glands

## Abstract

**Simple Summary:**

Skin derivatives, such as integumentary glands, keratin, and bony structures deriving from the epidermis or dermis, are commonly found in various vertebrates species and are suggested to possess a variety of functions that are closely related to the lifestyle of the species. However, most reptile glandular skin derivatives have undergone obvious degeneration due to environmental adaptation to dry land, with the exception of a few skin glands with specific functions. An oval organ/tissue called the dorsal gland spreading from the mid-cervical region to the anterior caudal region in the axial musculature on the dorsal mid-line has been reported in crocodilians, including *Alligator sinensis*, which is a critically endangered species of the 23 existing crocodilian species. Here, we present the first investigation to focus on particular states of cell proliferation and cell apoptosis, we provide direct molecular evidence which supports the speculation that it might serve as a holocrine secretory, as observed in the other known multicellular exocrine glands. Furthermore, the high-throughput analysis of gene-expression profiles at different timepoints of embryonic and neonatal *Alligator sinensis* suggested that it might function through the transport and deposition of pigment and lipids via lysosomal exocytosis, these function might relate to an adaptive function in the transition from an amniotic fluid environment to the terrestrial environment around hatching. The corresponding results have considerable importance in enriching our understanding of the intrinsic relationship between the skin derivatives and lifestyles of crocodilians. Furthermore, they have provided a theoretical basis for the rearing and management of newborn *Alligator sinensis*.

**Abstract:**

Significant advances have been made in the morphological observations of the dorsal gland (DG), an oval organ/tissue which lies on both sides of the dorsal midline of the crocodilian. In the current study, RNA sequencing (RNA-seq) was used to identify the changing patterns of *Alligator sinesis* DGs at different timepoints from the 31st embryonic day (E31) to the newly hatched 1st day (NH1). A comprehensive transcriptional changes of differentially expression gene (DEGs) involved in the melanogenesis, cholesterol metabolism, and cell apoptosis pathways suggested that the DG might serves as a functional secretory gland in formation, transport and deposition of pigment, and lipids secretion via lysosomal exocytosis. Furthermore, the remarkable immunohistochemical staining of proliferating cell nuclear antigen (PCNA) and B-cell lymphoma 2 (Bcl-2)-positive signals in the basilar cells, in parallel with the immuno-reactive TdT-mediated dUTP nick-End labeling(TUNEL) within suprabasal cells, provided direct molecular evidence supporting for the speculation that DG serves as a holocrine secretion mode. Finally, subsequent phylogenetic and immunohistochemical analysis for the PITX2, the identified DEGs in the RNA-seq, was helpful to further elucidate the transcriptional regulatory mechanism of candidate genes. In conclusion, the current results are of considerable importance in enriching our understanding of the intrinsic relationship between the skin derivatives and lifestyles of newborn *Alligator sinesis*.

## 1. Introduction

The integumentary glands of vertebrates have shown to be nearly exclusively particularistic in their orientation, this is can be partly credited to the amazing structural, chemical, and functional diversity shown in the adaptive microevolution of these structures [1]. Many glands deriving from the skin surfaces of vertebrates have functions in covering the body and isolating the external environment and also play a key role in many complex functions [2]. Contrary to the impression engendered by most textbooks, living reptiles possess a remarkable diversity of skin glands, which have been proven to be genetically related tissues but not homologous organs [3]. In crocodilians, two sets of integumental glands also known as musk glands, including cloacal glands and mandibular glands, are developed by an invagination of the lower layer of the epidermis [4]. The differences in the chemical composition and secretory granules structures of these skin glands have indicated that their roles may be different but that they are both related to pheromone communication between individuals [5]. In addition, several investigations among the crocodilian species have described an oval organ/tissue called the dorsal gland (DG), lying from the mid-cervical region to the anterior caudal region in the axial musculature on each side of the dorsal midline. For instance, the five and sixteen pairs of DGs have been reported in the nuchal region and the pectora pelvic regions in the Nile crocodile, respectively. In the *Alligator mississippiensis*, 20–22 pairs of DGs were first seen in an embryonic individual, where they were indicated by a rounded thickening and invagination of the stratum germinativum, which further migrated into the axial musculature or the inner surface of the dermis when reaching adulthood [6]. The Chinese alligator, *Alligator sinensis* also possesses DGs under the dermis adjacent to the muscle layer in the dermis of adults, juveniles, and a few embryonic individuals [7].

Although morphological evidence suggests that DGs are multicellular secretory glands, there is a lack of knowledge and explicit hypotheses regarding its function. For example, the DGs of *Alligator sinensis* were considered to be degenerate organs due to the absence of glandular pores attached to the skin’s surface [7]. However, the several layers of cuboidal columnar epithelium characterized by foamy cytoplasm with an abundance of mitochondria and short segments of rough endoplasmic reticulum have been observed in the peripheral lumen of DGs. Furthermore, electron microscopy has revealed a dense core or layering of hexagonal crystals, containing calcium, copper, iron, lead, potassium, and zinc, in both epithelial cells and secretory granules [4]. Although the morphological characteristics of the loose and laminated glandular wall are very similar to those of the musk glands, its small size and the absence of specific odor strongly indicates that the function of DG is likely to be different from that of the musk glands. For instance, crocodilian DGs have been suggested to function as rudimentary sensory organs similar to the lateral line system of fish [7]; meanwhile, the apparent paucity of nerves found in anatomical observations does not support the hypothesis that DGs are sensory organs [4]. In adult *Alligator mississippiensis*, considerable amounts of lipids have been found in secretory granules, and in parallel with the wide distribution over the dorsum, these lipids indicate that DGs might have function of keeping the scales in a good condition to make them comparable to the oil glands in the skin, as found in mammals [4]. During adaptation to terrestrial environments after hatching, the integumentary glands of amniote vertebrate including reptile improve their provision of mechanical resistance to water loss, microbical invasion, and mechanical resistance to wear and external agents. Therefore, the DG were collected around the timepoint of hatching to preliminarily elucidate the possible adaptive function in the transition from amniotic fluid environment to terrestrial environment, before and after hatching.

Holocrine secretion is a specific mode of secretion that involves the secretion of entire cytoplasmic materials with the remnants of dead cells, as observed in the multicellular exocrine glands of reptiles, birds, and mammals [8]. The function of DGs remains controversial; however, some scattered microscopical evidence, such as the occurrence of tight junctions and desmosomes between the interdigitation of epithelial cells lining the gland, have suggested that DGs give rise to the secretory product via holocrine secretion [4]. These morphological characteristics are similar to those of sebaceous glands (SGs) in birds, which produce a large amount of waxy fluid substance that is spread on feathers as a part of plumage maintenance [3]. It has been acknowledged that the predominant constituent cell population of SGs is lipid-producing sebocytes; these contribute 90% of skin surface lipids and possess various functions, such as antimicrobial peptide production, immunomodulation via lipids, hormone synthesis/metabolism, and the provision of an epithelial progenitor cell reservoir [9]. The holocrine process of sebum, derived from the sebocytes, begins with the proliferation of this undifferentiated and mitotically active population of peripheral, immature cells [10]; furthermore, the cells undergo nuclear degeneration and move more centrally, which can lead to the induction of apoptosis and the bursting of cells [11]. As the most important regulators of cell apoptosis, the key regulator of B-cell lymphoma 2 (Bcl-2) shows expression patterns in the uropygial gland; this indicates that the terminal differentiation of sebocytes during holocrine secretion in DGs is realized mainly through cell apoptosis [12]. Furthermore, as other central molecules responsible for DNA synthesis during replication [13], proliferating cell nuclear antigens (PCNAs) have been suggested to be associated with the survival and proliferation of sebocytes, resulting in the constant renewal of germinative layer cells throughout the life of DGs [14]. However, the states of cell proliferation and apoptosis in the DGs of crocodilians have not yet been reported on. It is necessary to further verify whether DGs function via the holocrine secretion; with this determination, we can provide a basis of information surrounding the glandular secretory mechanism, permitting the further exploration of its possible biological functions.

In the present study, we conducted immunohistochemistry (IHC) detection for cell differentiation and proliferation markers, including PCNA and Bcl-2, in parallel with the cell apoptotic assay using the TUNEL method; these were conducted to determine the particular states of cell differentiation and apoptosis in order to comprehensively verify whether the DG functioned via holocrine secretion or not. Furthermore, using a high-throughput RNA-seq, we aimed to identify the gene-expression profiles during different timepoints in the early development stages, especially before a distinctively recognizable form had been reached, to preliminarily elucidate their possible functions in the transition from an amniotic fluid environment to a terrestrial environment around the timepoint of hatching. Finally, due to the relatively scattered distribution of dorsal glands and the large number of adjacent tissues of dorsal glands, it was particularly necessary to exclude the interference of mRNA in non-DG tissues, such as skin, via the histochemical localization of candidate genes. To achieve this, the subsequently investigation focusing on the PITX2, that known as key transcription factors functioning in the vepidermal keratinocytes differentiation were designed, which would be helpful to further elucidate the transcriptional regulatory mechanism of DEGs. The corresponding results are of considerable importance in enriching our understanding of the intrinsic relationship between the skin derivatives and lifestyles of crocodilians and provide a theoretical basis for the rearing and management of newborn *Alligator sinensis*.

## 2. Materials and Methods

The animal protocols used in this study were in accordance with Measures for the Administration of the Permit for experimental animals provided by Ministry of Science and Technology of the People’s Republic of China (2nd ed. No. 593, 2001). Special care was taken in following the Guide for the Care and Use of Laboratory Animals, which prevents or minimizes the stress of animals in all stages of experiments and is also be approved by the Academic Ethics Committee of Anhui Normal University (Project identification code: No. AHNU-ET2021008; data of approval: 6 March 2021).

### 2.1. Animal Management and Samples Collection

Ten *A. sinensis* eggs from the same clutch were collected on the 15th days of incubation from Wuhu Dajiang Farmer (Wuhu, Anhui, China; longitude 118.41°, latitude 31.29°). After the preliminary detection of fertilization status, all the eggs were carefully transferred to the Alligator Normal University for artificial incubation using incubation boxes at the temperature of 29 °C and humidity of 90%. Since the dorsal gland is considered to belong to the skin gland [7], eight of ten eggs were sampled from 21 to 25 stages, which correspond to embryonic 31 day (E31), 36 day (E36), 41 day (E41), and 45 day (E45), covering scale occurrence and skin color formation, according to the external morphological description of 28 stages in the embryonic *A. sinensis* [15]. Additionally, when developed to the newly hatched 1st day (NH1) stage, the remaining two individuals were selected as the last groups of the study, based on the finding that the DG at this developmental stage is beginning to approach the adult structure [7]. For each timepoint, all the individuals were euthanized via decapitation after deep anesthesia with an intraperitoneal injection of sodium pentobarbital (50–100 mg/kg, Propbs, Beijing, China). According to the procedure described in the adult *A. mississippiensis* [4], after the removal of the dorsal skin from the mid-cervical to the anterior caudal regions, the DG was exposed by probing through or scraping away the skeletal muscle and connective tissue on the underside of the dermis. All the DG samples located in the mid-cervical region, pectoral, and anterior caudal regions from the left side of mid-line were fixed with 4% paraformaldehyde in 0.1 M phosphate buffer for morphological study, and the DG samples collected from the right side of mid-line were stored at liquid nitrogen for RNA isolation and transcriptome sequencing.

### 2.2. Hematoxylin-Eosin (HE) Staining

Once they were deparaffinized, all the DG samples were passed through a rehydration series from 100% to 70% ethanol; they were then embedded in paraffin and serially sectioned at 6 μm. In preparation for HE staining, based on the differences between the pectoral caudal, mid-cervical, and anterior caudal regions, the slides of DGs that showed more adhesion with the connective tissue on the underside of the dermis were selected from pectoral caudal regions. Additionally, the principles of slide selection for immunohistochemistry IHC and TUNEL assay were followed. According to the magnification procedure, HE staining was performed using a hematoxylin-eosin (HE) staining kit (NO. E607318, Sangong, China) according to the following procedure: Briefly, the slides were washed in distilled water and the nuclei was stained in hematoxylin staining solution (Component A) for 5~10 min. Then, the samples were rinsed in running tap water for 5 min and were differentiated with 1% hydrochloric acid-ethanol for 10~30 s and rinsed in running tap water for 1 min. Following bluing in PBS/PBST for 30 s^−1^ min, the slides were rinsed in running tap water for 5 min, washed in 95% alcohol for 5~10 s, and counter-stained using eosin staining solution (Component B) for 30 s^−2^ min. Finally, the slides were dehydrated through 95% alcohol and cleared in xylene for 5 min. Micrographs of the sections were taken using a bright-field microscope (Zeiss, Germany).

### 2.3. RNA Isolation, Library Construction, and Sequencing

Equal amounts (100 mg on average) of DG samples from the right side of the midline were pooled for cDNA library construction and numbered as E31-1, E31-2, E36-1, E36-2, E41-1, E41-2, E45-1, E45-2, NH1-1, and NH1-2. Samples (50 mg on average) per pooled individual were homogenized for total RNA isolation using RNAprep Pure Tissue Kit (Tiangen, China), followed by incubation with DNaseI (80 μL/per sample) for 15 min at room temperature to remove the genomics DNA contamination. The RNA concentration and quality were determined by a NanoDrop 2000 Spectrophotometer (ThermoFisher, Scientific, California, USA). The integrity of the RNA from the pooled samples was determined using an Agilent 2100 Bioanalyzer and RNA electrophoresis. The corresponding results of RNA quality control are shown in Figure S1 and Table S1. Ten cDNA libraries were constructed through the mRNAs enrichment using magnetic beads with Oligo (dT) and then fragmented into small pieces with NEBNext first-strand synthesis reaction buffer. First- and second-strand cDNA samples were synthesized with random hexamer primers using M-MuLV Reverse Transcriptase Buffer and DNA Polymerase I and RNase H, respectively. Using the Qiaquick PCR extraction kit, the cDNA was purified and enriched by PCR amplification to obtain suitable fragments. After removing the adapter sequences and low-quality reads (Q < 20), an average of 70.87, 78.43, 80.81, 60.14, and 65.48 million raw reads were obtained from the E31, E36, E41, E46, and NH1 cDNA libraries, respectively (Table S2).

After sequence mapping—aligned to the *A. sinensis* genome sequences using TopHat v2.0.9—the clean reads of the E31 (61.26 million; 84.04%), E36 (67.97 million; 86.83%), E41 (69.10 million; 85.51%), E45 (52.11 million; 86.65%), and NH1 (56.50 million; 86.17%) samples were successfully mapped, allowing no more than two base mismatches (Table S3). The read counts of each gene were summarized by HTSeq v0.6.1 and adjusted using the edgeR program package through one scaling-normalized factor. The mRNA length and sequencing depth were homogenized using reads per kilo, using a gene per million reads (RPKM) algorithm to calculate the gene expression. Then, the differential expression genes (DEGs) were assessed using the likelihood ratio tests in the edgeR package in R language using the threshold value of |log2 (Fold change)| ≥ 1 with FDR-adjusted *p*-value ≤ 0.01. The GO-enrichment analyses for DEGs in different groups were performed using the GOseq R package, in which a corrected *p*-value ≤ 0.05 was considered to indicate significant enrichment by DEGs. Here, the enrichment score for each cluster was calculated as the negative log of the geometric mean of the *p* values in the cluster.

### 2.4. Quantitative Real-Time PCR (RT-qPCR)

The data of the RT-qPCR were obtained from two biological and three technical replicates. Firstly, the sub-samples of total RNA extractions from the E36, E41, and NH1 samples were selected and were reverse-transcribed into cDNAs using the PrimeScript RT Master Mix kits (Takara Biomedical Technology, Beijing, China) as follows: a total of 2 μg RNA was treated with gDNA Eraser for 2 min at 42 °C for genomic DNA removal; then, it was reverse-transcribed using Master Mix at 37 °C for 15 min, followed by 85 °C for 5 s. Then, further RT-PCR analyses were performed according to the instruction manuals of SYBR Premix Ex TaqII Kit (TaKaRa, Japan), as follows: the amplification reactions containing 1.6 μL of cDNA, 10 μL of SYBR green ER master mix, 7.6 μL of nuclease-free water, and 0.4 μL of forward and reverse primers were conducted at 95 °C for 5 min for 1 cycle, followed by 38 cycles, including 94 °C for 40 s, 56 °C for 60 s, 72 °C for 90 s, and extension for 10 min at 72 °C. The primers were designed using Premier 5.0, the corresponding information—including GenBank accession numbers and paired-primers sequences for target and internal control genes—are listed in Table S4, and the designed primers were all validated using melting curves, which showed that the amplification efficiencies ranged from 94.3 to 103.4%. The relative gene expression levels were calculated using the 2^−ΔΔ^CT method with Ribosomal protein L8 (RPL8) as an internal reference gene [16].

### 2.5. Immunohistochemistry (IHC)

For the IHC analyses, the slides were hydrated and blocked in normal fetal bovine serum for 30 min following deparaffinization. After being naturally cooled to indoor temperatures, the sections were incubated at 4 °C overnight with the commercial primary antibody of anti-PCNA (bs-2006R, 1:300), anti-Bcl-2(bs-0032R, 1:250), and anti-PITX2 antibody prepared by the recombinant prokaryotic expression system at dilution of 1:400. Negative controls were incubated with a non-specific anti-rabbit IgG. After washed in the PBS for at least three times, the sliders were incubated with horseradish peroxidase-labeled species-appropriate secondary antibodies (P0202, Beyotime Biotechnology, Shanghai, China) at a dilution of 1:100 for 30 min at room temperature. Following additional washing, the sliders were treated with 0.2 mL color-substrate solution for 10 min according to the operating instruction of DAB Horseradish Peroxidase Color Development Kit (P0202, beyotime, China). After that, all the slides were immediately counterstained with hematoxylin for 2 min and further washed using tap water. After deparaffinization in 70% to 100% ethanol gradient and xylene, the slides were covered with a cover glass for further photographing using a light microscope. Negative control sections were processed simultaneously, as described previously without adding an antibody but using an equal volume of PBS instead.

### 2.6. TdT-Mediated dUTP Nick-End Labeling (TUNEL) Assay for Cell Apoptosis

The cell apoptotic analysis was performed on three slides randomly selected to be technical replicates from the E36, E41, and NH1 using commercial TUNEL assay kit (C1089, Beyotime, China). According to the manufacturer’s instructions, all selected sections were pre-treated with 20 mg/mL Proteinase K for 15 min at 37 °C, washed in PBS and then incubated with the TUNEL reaction mixture for 1 h at 37 °C. After that, two investigators examined the samples microscopically in a blinded fashion under a scanning microscope (Zeiss, Oberkochen, Germany) with 488 nm excitation and 530 nm emission to further determine the apoptosis rate based on the percentage of the TUNEL-positive cells.

### 2.7. Molecular Cloning and Phylogenetic Analysis of PITX2 cDNA

RNA extraction and reverse transcription were performed following the manufacturer’s protocol as previously described. The complete coding region sequences within the *PITX2* cDNA of *A. sinensis* were obtained by polymerase chain reaction (PCR) according to the highly conserved regions using the primers (Forward: 5′-TTGCCCATGAACTGCATG-3′; Reverse: 5′-GAATATCTCACACGGGTC-3′) through polymerase chain reaction. The resultant PCR products were purified, cloned, sequenced, and confirmed by BLAST, in accordance with the procedures described in detail in our previously work [16]. The deduced amino acid sequences were aligned using the ClustalX2 program. Any gaps were removed from the sequence using the BioEdit program. The molecular phylogenetic analysis for full-length amino acid sequences of the *PITX2* were constructed in MEGA7 using the maximum likelihood method. Initial tree(s) for the heuristic search were obtained automatically by applying neighbor-joining algorithms to a matrix of pairwise distances estimated using a JTT model and then selecting the topology with superior log likelihood value. The information of other organisms’ PITX2 sequences are shown in Table S5.

### 2.8. Statistical Analysis

Statistical were performed before data normality analysis using Kolmogorov–Smirnov goodness-of-fit test, with the SPSS statistical software program (version 19.0; SPSS Inc., Chicago, IL, USA).

## 3. Results

### 3.1. Morphology Characteristics of DG during Different Developmental Stages

**Figure 1:**
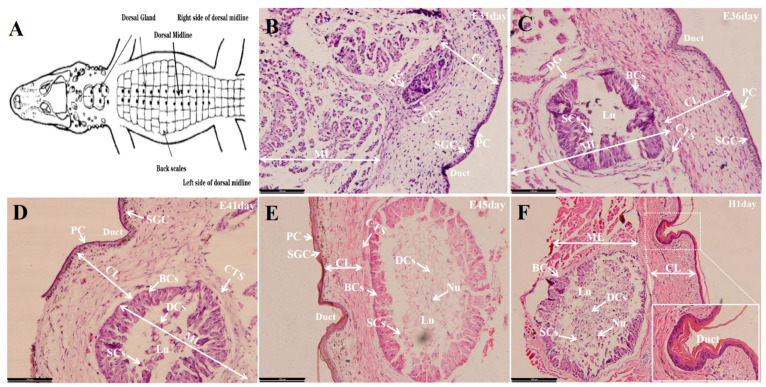
(**A**) Schematic Diagram of DG location of *A. sinesis*. Morphological descriptions of DG collected from different developmental stages including E31 (**B**), E36 (**C**), E41 (**D**), E45 (**E**), and NH1 (**F**) days. DG: Dorsal gland, PC: Periderm cells, SGC: Stratum germinativum cells, CTS: Connective-tissues sheath, ML: Muscle layer; CL: Corium layer; BCs: Basilar cells, SCs: Secretory cells, DCs: Degenerative cells, Lu: Lumen, Nu: Nuclei.

As illustrated in Figure 1, two well-defined types of cells, periderm cells (PC), with flattened nuclei located in the outer layer, and stratum germinativum cells (SGC), characterized by large spherical nuclei located adjacently to the corium layer (CL), were present throughout E31 and subsequent developmental stages. As early as the E31, DG was observed as a rounded thickening and dense mass of cells, which was characterized as looser and more scattered towards the center, nearly extending through the CL to the outer edge of the underlying muscle layer (ML). At E36, DG began to invaginate into the ML and divide into periphery arrangement basilar cells (BCs), showing large oval or spherical nuclei. Within the irregular central lumen (Lu), a large number of secretory cells (SCs), characterizing a large part of the granular contents, were observed as having a looser arrangement than that of the BCs. When further developing to the E41 and E45, the morphological characteristics of the peripheral BCs and central Lu in the DG were similar to those in E31, except for a mass of irregular and elongated cells characterized by a pyknotic nuclei (Nu) and empty cell membranes. These degenerative cells (DCs) might be the remains of cells from which the granular or the lipids secretion were emptied. Additionally, the DG collected at NH1 was beginning to approach an adult structure. The size of Lu was several times larger than it was at the preceding stages. Additionally, in the stratum germinativum and subjacent CL, a greater number of and denser pigment granules appeared than those in the earlier stage.

### 3.2. Identification of Differentiated Expressed Genes (DEGs)

**Figure 2:**
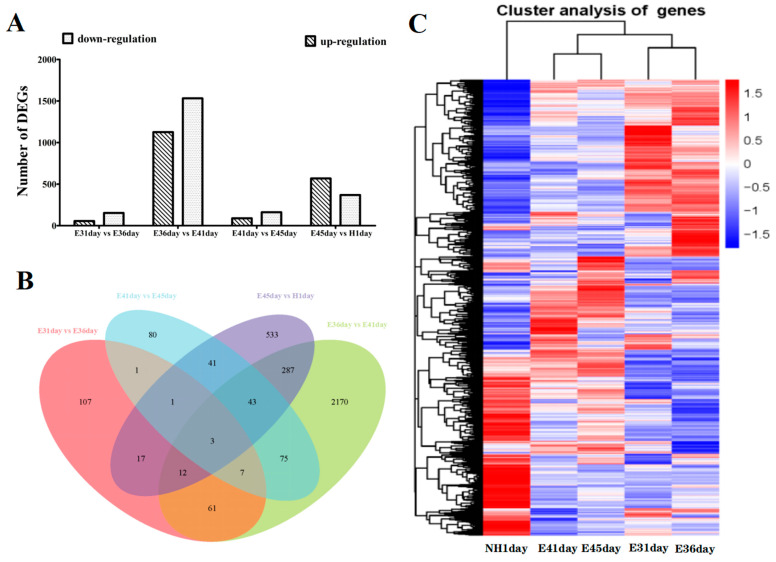
Identification of differentiated expressed genes (DEGs) between E31, E36, E41, E45, and NH1 cDNA libraries in *A. sinensis*. (**A**) Number of DEGs identified through near-term pairwise comparisons. (**B**) Venn diagram showed the number of DEGs in different groups. (**C**) Heatmap diagram of the expression patterns of DEGs between different groups. The color of red and green represent up-regulated and down-regulated genes, respectively.

As illustrated in Figure 2A, a total of 4055 DEGs were identified through near-term pairwise results, which referred to two consecutive timepoint comparisons, among these, 209, 2658, 251 and 937 DEGs were found between E31 vs. E36, E36 vs. E41, E41 vs. E45, and E45 vs. NH1, respectively. Compared with the E31 group, 56 and 153 genes were identified to be up- and down-regulated in the E36 group. The comparison of the E36 revealed 1126 genes that were up-regulated and 1532 genes that were down-regulated in the E41 group. A total of 89 and 162 genes were up- and down-regulated in E45 compared with the E41, respectively. A total of 568 up-regulated and 369 down-regulated genes were identified in the NH1 compared with the E45. Of the 209 DEGs between E31 and E36, 83 were found to be differentiated expressed in comparison with those between E36 and E41. Of 83 genes, 12 were differentially expressed between E31 and E36 and between E41 and E45. Of the 251 up-/down-regulated genes, 88 genes between E41 and E45 were found to be differentially expressed in comparison with those between E45 and NH1 (Figure 2B).

As visualized by results presented in the heatmaps (Figure 2C), the expression patterns within E31 and E41 were more closely related to E36 and E45, respectively. Moreover, most of the DEGs displayed great differences in the expression profile from the NH1 compared with that of E31/E36 and E41/E45. Based on the results of hierarchical cluster analysis, the gene transcript profiles of E36, E41, and NH1 were selected for subsequent analysis.

### 3.3. Functional Classification by Gene Ontology and KEGG Pathway Analysis

**Figure 3:**
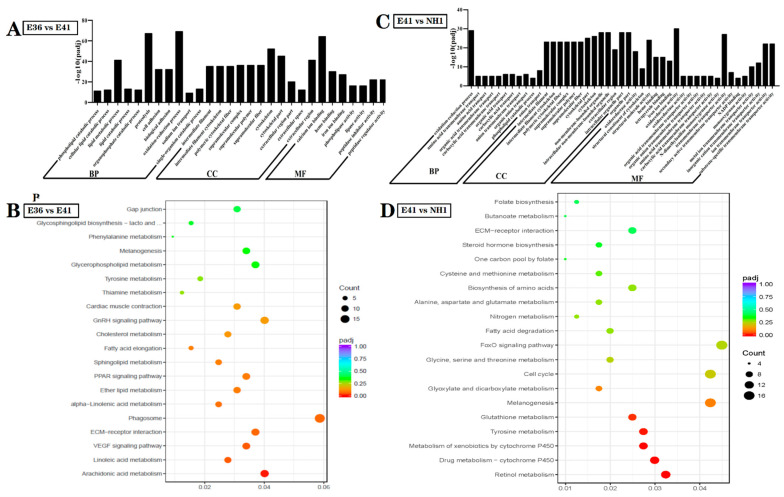
(**A**,**B**) The top 30 most enriched gene ontology (GO) terms (*p*-value ≤ 0.05) of DEGs between E36 vs. E41 and E41 vs. NH1 belonging to biological processes, cellular components, and molecular functions, respectively. (**C**,**D**) DEGs between E36 vs. E41 and E41 vs. NH1 enriched in KEGG pathways. The dot size indicates the number of DEGs of the pathway, and the dot colour indicates the adjusted *p*-value.

The proportions of enriched genes in the E36 vs. E41 comparison were summarized into three main GO categories, such as biological process, cellular component, and molecular function (Figure 3A). In the biological process category, the dominating GO terms enriched in the E36 vs. E41 comparison were predominantly related to “proteolysis”, “oxidation-reduction process”, and “lipid metabolic process”. Additionally, “cytoskeletal part” and “cytoskeleton” were significantly enriched in the cellular component category. In the molecular function category, most DEGs were involved in some functional categories, such as “calcium ion binding”, “heme binding”, “peptidase inhibitor activity”, and “peptidase regulatory activity”.

The most significantly enriched GO term in the DEGs between E41 and NH1 was the “oxidation-reduction process” that was subject to biological processes. In the cellular component category, the dominating GO terms were “cytoskeleton”, “intracellular non-membrane-bounded organelle”, “intracellular non-membrane-bounded organelle”, and “ intracelullar organelle”. In the molecular function category, the dominant GO terms were “structural molecule activity”, “oxidoreductase activity”, “transmembrane transporter activity”, and “ion transmembrane transporter activity” (Figure 3B).

To further explore the biological functions of the DEGs, an enrichment analysis based on the KEGG database was performed. The top 20 pathways for the most prominent DEGs are listed in Figure 3C,D. Among the 2658 DEGs identified in the comparison between E36 and E41, the major enriched pathways were related to “peptidase activity”, “calcium ion binding”, “cytoskeleton”, “oxidation-reduction process”, “proteolysis”, and “lipid metabolic process”. Additionally, the KEGG pathway analysis based on the differential expressed genes between E41 and NH1 revealed that the major enriched pathways were “oxidation-reduction process”, “oxidoreductase activity”, “transmembrane transporter activity”, “ion transmembrane transporter activity”, “organelle part”, and “non-membrane-bounded organelle”. The diagrams showed the relative expression levels of total DEGs among different groups.

### 3.4. Critical DEGs Involving in the Melanogenesis, Cholesterol Metabolism and Cell Apoptosis

From the NGS-based RNA-Seq analysis (Table 1), a list of the DEGs between E36 vs. E41 and E41 vs. NH1 were found to be related to the pathways of melanogenesis (asn04916), cholesterol metabolism (asn04979) and cell apoptosis (asn04210). For instance, 11 DEGs involved in the cholesterol metabolism pathway were identified, and four genes, including *ABCA7*, *SOAT1*, and *LRP1*, were up-regulated in E41 compared with E36 (*p* < 0.01), and there was no significant difference between E41 and NH1 (*p* > 0.05). However, three genes, *PCSK9*, *APOA-1*, and *TSPO,* were specifically up-regulated in E41 in comparison with NH1 (*p* < 0.01), and there was no significant difference between E36 and E41 (*p* > 0.05). Additionally, four genes, *APOB*, *LRP2*, *APOC3*, and *LIPG,* were identified as significant down-regulated in E36 and NH1 but significantly up-regulated in E41 (*p* < 0.01).

Additionally, fifteen genes involved in melanogenesis pathway were identified, including four genes, *HMG*, *FZD7*, *ADCY3*, and *PLCB3,* which were identified as being up-regulated in the E41 compared with E36 (*p* < 0.01), whereas there was no significant difference in these genes between E41 and NH1 (*p* > 0.05). However, the expressions of *DCT*, *Try*, *MC1R*, *TYRP1*, *WNT4*, *WNT16*, and *WNT6* were lower in the NH1 compared with those in E41, but these were similar between E36 and E41 (*p* > 0.05). Additionally, the expression of four genes, *Kitl*, *WNT5a*, *FZD10,* and *FZD1,* were specifically up-regulated in E41 day compared with those within the E36 and NH1 (*p* < 0.01).

For the cell apoptosis pathway, five genes including MCP1, α-tubulin 3, CRLM2, CSF2RB, and BIRC5, were down-regulated in the E41 compared with those within the E36 (*p* < 0.01), whereas no significant difference was detected between E41 and NH1 (*p* > 0.05). However, *APOC1*, *CTSW*, *α-tubulin8*, *FAS*, ITPR2, and Bcl2 were up-regulated in NH1 compared with those within the E41, no significant difference was detected between E36 and E41 (*p* > 0.05). Additionally, the expressed of GPX2 were specifically up-regulated in the E41 day compared with those within the E36 and NH1 (*p* < 0.01).

### 3.5. Expression Profiles Analysis by RT-qPCR

**Figure 4:**
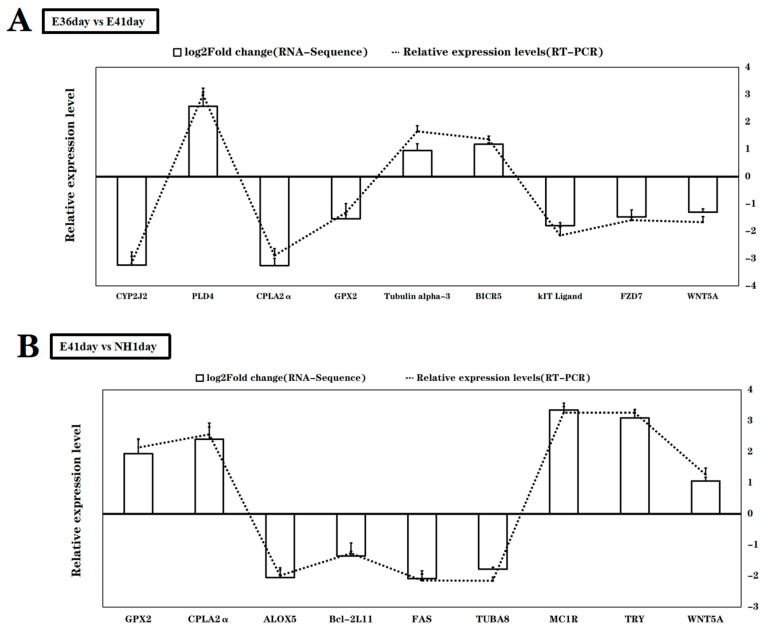
Expression validation of selected DEGs identified between E36 vs. E41 (**A**) and E41 vs. NH1 (**B**) via the relative expression detecting by RT-qPCR (Bar) compared with the transcript abundances from RNA-Seq (lines, the values were calculated by the read counts calculated by FPKM method). FZD7: frizzled class receptor 7; Kitl: kit ligand; WNT5A: wingless-type integration site member 5A; MC1R: melanocortin 1 receptor; BICR5: baculoviral IAP repeat containing 5; TUBA8: tubulin alpha-8; TUBA3: tubulin alpha-3; FAS: Fas cell surface death receptor; CPLA2α: cytosolic phospholipase A2 epsilon; PLD4: phospholipase D member 4; CYP2J2: cytochrome P450 2J2; Alox5: arachidonate 5-lipoxygenase.

To validate the differential expression patterns of DEGs between E36 vs. E41 and E41 vs. NH1, 12 genes were randomly selected for RT-qPCR analysis to evaluate the accuracy of DGs provided by the RNA sequencing based on the read counts calculated by the FPKM method. These selected genes included four melanogenesis-associated genes (*KIT ligand*, *FZD7*, *WNT5A*, *TYR,* and *MC1R*), four apoptosis-related genes (BIRC5, Tubulin α-3, FAS, and BCL-2), and five genes involved in cholesterol metabolism, such as *CPLA2α*, *PLD4*, *CYP2J2,* and *ALOX5*. Our results revealed that the expression patterns of all the selected genes were in agreement with the RNA-sequencing transcript and further indicated the high accuracy of the DEGs assessment obtained from RNA sequencing (Figure 4).

### 3.6. Immunolocalization of PCNA, Bcl-2, and TUNEL-Positive Apoptotic Cells

**Figure 5:**
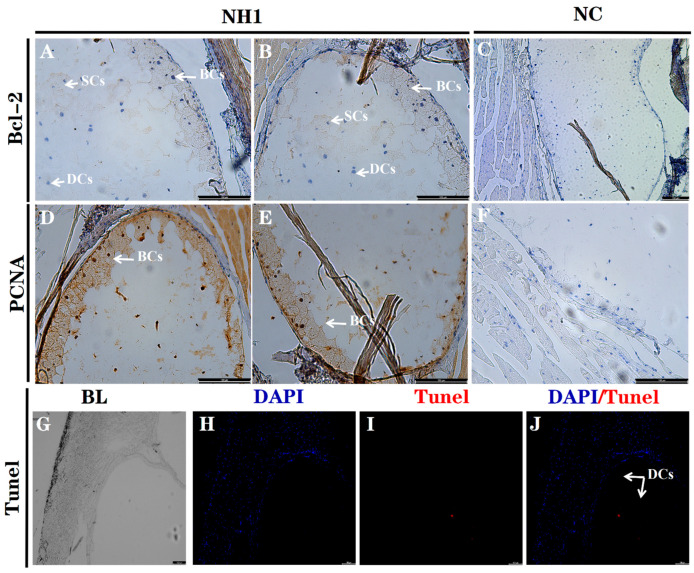
Result of particular states of cell differentiation and apoptosis of DG collected at NH1 when approach to the adult structure. (**A**–**F**): Immunohistochemical staining of Bcl-2 ((**A**,**B**); Bar = 150 μm), PCNA ((**D**,**E**); Bar = 150 μm), and negative control ((**C**,**F**); Bar = 150 μm) in DG tissues collected from the NH1. (**G**–**J**): TdT-mediated dUTP Nick-End Labeling (TUNEL) assay for cell apoptosis within the DG (Bar = 100 μm). The blue immunosignal indicates DAPI staining nuclei and the red color denotes TUNEL labeled signals. BCs: Basilar cells; SCs: Secretory cells; DCs: Degenerative cells; NC, negative control.

This is the first report focusing on particular states of cell differentiation and apoptosis of DG in the crocodilian species. Our current data indicated that remarkable PCNA and Bcl-2 positive-signals were found in the cytoplasm and nucleus of the BCs, whereas the DCs showed negative reaction to the corresponding antibodies, although a few cell nuclei in the SCs exhibited a positive reaction (Figure 5A,B,D,E). However, no specific immunoreactivity was detected in the negative control of Bcl-2 (Figure 5C) and PCNA (Figure 5F). Additionally, the specific TUNEL-positive signals appearing in the irregular central Lu were suspected to be located within the DCs, which suggested that the DCs undergo apoptosis (Figure 5G–J).

### 3.7. Molecular Cloning and Phylogenetic Analysis of PITX2

**Figure 6:**
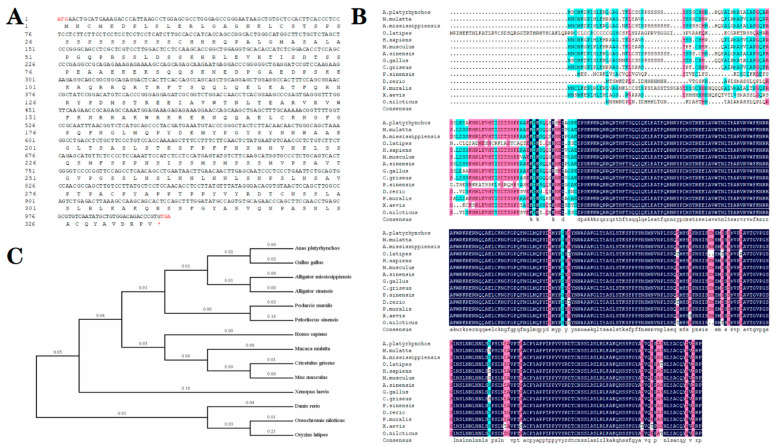
(**A**) The nucleotide sequence and the deduced amino acids sequence of *A.sinensis* PITX2, an asterisk (*) indicates the stop codon. (**B**) Multiple sequence alignment of PITX2 amino acid sequence. Light shaded color represented identity of the amino acid residues more than 75% among various species. (**C**) Phylogenetic tree of vertebrate PITX2 amino acid sequences using the maximum likelihood method.

As illustrated within the Figure 6A, a 1008 bp PITX2 cDNA of *A. sinensis* was obtained and submitted to GenBank (Accession No. MK992783), which included a complete ORF which encoded a precursor protein of 335 deduced amino acids. As a result of the alignment analysis of deduced amino acids sequences illustrated in the Figure 6B, the PITX2 of *A. sinensis* has a high identity with *A. mississippiensis* (99.7%), *Anas platyrhynchos* (95.2%), and *Gallus gallus* (94.1%). The molecular phylogenetic analysis of the PITX2 amino acid sequences using the maximum likelihood method indicated that crocodilians were more closely related to birds, reflected by the formation of clusters with those species such as *Anas platyrhynchos* and *Gallus gallus*, and were also grouped with turtles, including *Podarcis muralis* and *Pelodiscus sinensis* (Figure 6C).

### 3.8. Distribution of PITX2 within Brain and Peripheral Tissues

**Figure 7:**
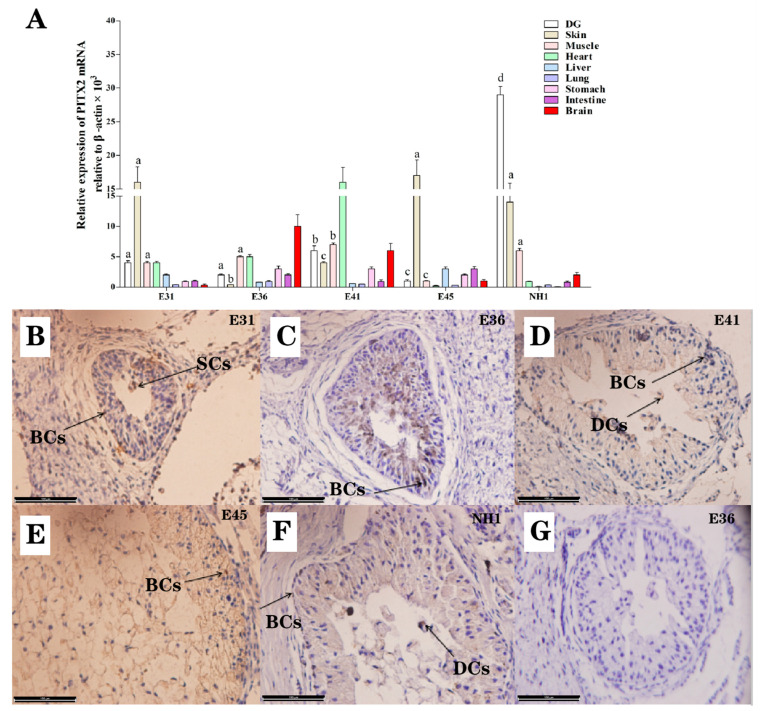
(**A**) Relative expression of *PITX2* mRNA in different tissues of *Alligator sinensis*. Data are the Mean ± SD. Different letters above the bars represent significant differences between different developmental stages. (**B**–**G**) The immunohistochemical staining of PITX2 and negative control ((**C**,**F**); Bar = 150 μm) in DG tissues collected from E31 (**B**), E36 (**C**), E41 (**D**), E45 (**E**), and NH1 (**F**). Negative control was conducted also treated with normal rabbit serum instead of primary antisera (**G**). BCs: Basilar cells, SCs: Secretory cells, DCs: Degenerative cells.

As illustrated within the Figure 7A, the expression of PITX2 mRNA in the DG, skin, muscle, heart, liver, lung, stomach, intestine, and brain of *A. sinensis* collected from E31, E36, E41, E45, and NH1 were investigated by qPCR. The mRNA abundances of *PITX2* were relatively higher in the DG, skin, and muscle than that of other peripheral tissues, such as in the liver, lung, stomach, intestine, and brain (*p* < 0.05). It has been demonstrated that the mRNA expression of *PITX2* in the DG remained constant from the E31 to E36 after being rapidly up-regulated in E41, and its mRNA abundance significantly decreased and finally reached peak value at NH1 (*p* < 0.05). The IHC results of PITX2 showed that the moderately abundant PITX2-positive signals were mainly observed in SCs within the DG collected from E36, whereas weak PITX2 was observed to be expressed within the BCs and SCs collected from the E41 and NH1 stages (Figure 7B–F).

## 4. Discussion

Epidermal glands are specialized multicellular organs in circumscribed body areas that exhibit holocrine secretion, which has been suggested to be a specific secretion mode of entire cytoplasmic materials with the remnants of dead cells. As observed in the multicellular exocrine glands of chicken uropygial glands, the sebum produced by sebocytes undergo holocrine secretion, in which the entire plasma membrane and the entire limiting membrane of secretory vesicles break down to secrete all the intracellular materials [3]. The holocrine secretion process begins with the proliferation of peripheral immature cells, which are characterized as mitotically active; this is followed by the development of the basial layer, which leads to the induction of cell apoptosis and bursts during maturation [11]. Electron microscopy indicated that the degeneration of the epithelial cells in the DG lumen lyses the secretory product via a holocrine secretion mode in the crocodilian species [6]. However, there is no other direct molecular or cellular evidence derived from particular cell-differentiation states or programmed cell death and cell apoptosis. The proliferating cell nuclear antigen (PCNA) is suggested to be the one of the central molecules responsible for the decisions of the life and the death of a cell [17]. Additionally, as a member of the inhibitor of apoptosis protein families, B-cell lymphoma-2 (Bcl-2) has been suggested to play a crucial role in the balance between cell survival and cell death [18]. In the present research, a TUNEL assay—accompanied by PCNA and Bcl-2 immunohistochemical staining—was performed to investigate the state of cell proliferation and apoptosis in the crocodilian DGs collected at NH1 (the stage approaching the adult structure). This is consistent with the specific expression patterns of survival factors which occur in the specialized holocrine secretory gland, which has been reported to be in the avian uropygial gland [12]. The positive immunoreactivity for PCNA and Bcl-2 were mainly detected within the BCs, whereas the suprabasal layers undergo apoptosis; this is because the related TUNEL-positive signals were specifically observed in the secretory and/or degenerative cells, suggesting that these topologically distinct compartments of cell apoptosis and proliferation occur in the DGs of crocodilian species. This is the first report focusing on the particular cell differentiation and programmed cell death or apoptosis of DGs in the crocodilian species; therefore, we further provide direct evidence supporting the idea that DGs serve as holocrine glands, similarly to the uropygial glands of birds.

Programmed cell death plays important roles in the homeostasis and function of skin and its appendages [19]. As the major constituent of microtubule structure, the polymerization alterations of tubulin leads to visible changes in the response of microtubule structures to DNA damage [20]. For instance, the reversible α-tubulin acetylation influences microtubule structures, which are subsequently involved in cellular apoptosis and autophagy [21]. However, the declining α-tubulin acetylation coordinates the structure and function of microtubules and further performs critical roles in cell mitosis and chromosome segregation, as well as in cell migration [22]. Furthermore, the interaction partners of antiapoptotic protein p35 are suggested to be colocalized with a-tubulin in the cytoplasm in the absence of a death stimulus [23]. In the present study—although the near-term pairwise comparisons among E36, E41, and NH1 were not identical—Tubulin α-3 and Tubulin α-8 (belonging to the tubulin family) both showed the lowest expression levels in E41, in which a mass of DCs showing pyknotic Nu and empty cell membranes were observed. Thus, we speculated that the holocrine secretion induced by programmed apoptosis during DG development may be related to the microtubule structure regulation via tubulin family members. Moreover, the other genes involved in the cell apoptosis pathway, such as *CLDN-1*, *GPX2*, and *APOC1*, were identified to be DEGs at different developmental stages (Table 1). For instance, as the mediator of the barrier leakage of the tight junction (TJ), claudin-1 (CLDN-1) is suggested to be necessary for the accumulation of degenerated sebocytes in sebaceous ducts, which exhibit the form of a polarized stratified epithelium equipped with TJ [8]. Additionally, the apolipoprotein C-1 (APOC1) impairs α-tubulin acetylation and further inhibits apoptosis through the inactivation of caspase-3 [24]. In addition, the glutathione peroxidase family member GPX2 plays an important role in the defense against oxidative stress by scavenging hydroperoxides and free radicals [25]. Furthermore, it has been proposed that GPx-cDNA-transfected cells are more resistant to apoptosis in a p53-dependent manner [26]. The evidence as a whole suggested that these genes are closely related to the cell apoptosis response in controlled self-destruction during holocrine secretion in DGs.

Melanocytes are pigment-producing cells which synthesize the melanin that governs skin and hair color, and which protects individuals from environmental stimuli. For reptilians, the multicellular and, especially, circumscribed epidermal glands are suggested to play a major role in pigmentation or a morphological role in the color change of the shell and the skin. As proposed for the American alligator, the pigmentation of the epidermis of embryos is mostly derived from epidermal melanocytes [6]. The epidermal melanocytes are distributed in small areas of the epidermis, along the margins and center of the scales, which are responsible for the spot-like pigmentation of scales in young *Crocodilus niloticus* [27]. The kit-ligand is thought to be indispensable for the early phase of melanocyte development both in vivo and in vitro [28]. The membrane-bound kit-ligand captures cell-surface-expressed c-kits to provide a survival signaling mechanism for the melanocyte homeostasis of mice [29]. Moreover, mutations in kit-ligands influence the melanocyte morphogenesis, including cell communication, signal transduction, and cell migration. For instance, the kit-ligand α mutants in Zebrafish 4 days post-fertilization retain 60% of their wildtype embryonic melanocyte but fail to migrate or undergo programmed cell death [30]. In the present study, the expression of *kit-ligands* increased significantly from E36 to a peak at E41 and then decreased significantly at NH1. The up-regulated expression of *kit-ligands* was synchronized with pigmentation, which became more some diffuse and very faint, particularly on the forehead, jaw, and back at the E41 timepoint (Figure S2); this suggests that the specific expression pattern of the *kit-ligand* may be related to the migration of the spot-like pigmentation of scales during embryonic development. This suggestion was consistent with those surrounding most phylogenetically related species, such as birds, in which the kit signaling system is suggested to play a key role in melanocyte development and epidermal homeostasis via melanocyte migration and proliferation [12]. In addition, the dimerization-defective mutant of the melanocortin-1 receptor (MC1R) both inhibits melanin synthesis and enhances cell migration in human melanoma cells [31]. In mammalians, melanin biosynthesis is mainly catalyzed by tyrosinase-related protein-1 (*TRP1*), which has been suggested to be a modifier of the melanogenic pathway and other melanocyte functions [32]. The non-canonical Wnt molecule of WNT5A has been suggested to be involved in melanocyte proliferation and melanogenesis through the down-regulation of the pigment-cell-specific genes of frizzled-class receptor members (FZDs) and tyrosinase (TYR) [33]. In the present study, along with a number of differentially expressed melanogenesis-associated genes (such as *MC1R*, *TYR*, *TRP1*, *HMG*, and *ADCY3*), the frizzled-class receptor members (including *FZD1*, *FZD6*, *FZD7*, and *FZD10*) and Wnt signaling molecules (such as *WNT5A*, *WNT4*, *WNT6*, and *WNT6*) were observed to be significantly up-regulated at E41, in comparison with both E31 and NH1. The corresponding results indicated that the development and function of DGs might be related to the formation, transport, and deposition of pigment production via melanin.

Recently, there have been a few studies focusing on the discharge secretions, which have proposed hypotheses on the function of DGs. For example, the authors of [6] suggested that considerable amounts of lipids and glycoproteins might be present in both the epithelial lining cells and secretory products; this hypothesis was based on the anatomy and histochemistry characteristics uncovered through Sudan Black B staining. Similarly, the scattered fat- or oil-filled masses in the DGs of *Alligator mississippi* were suggested to consist of fat droplets deriving from the broken-down cells located in the lumen [4]. Materials including proteins, lipids, polysaccharides, and nucleic acids can reach lysosomes through different forms of endocytosis and can further release their content extracellularly via lysosomal exocytosis [34]. The intracellular application of cytosolic phospholipase A2-epsilon (cPLA2α) leads to an increase in the number of granules available for release and requires the combined actions of arachidonic acid and lysophosphatidylcholine [35]. Additionally, as a receptor-regulated signaling enzyme involved in exocytosis, phagocytosis, actin dynamics, and membrane trafficking-phospholipase D (PLD) is considered to be essential for the stimulated degranulation of cells via the membrane lipid environment alteration process, which is achieved through fatty acids [36]. Furthermore, Sterol O-Acyltransferase 1 (SOAT1), a regulator of cholesteryl ester formation, has been proven to be functional in maintaining readily releasable cholesterol reserves [37], as well as being necessary for the mobilization of stored ester pools and the generation of free cholesterol for adrenal steroidogenesis [38]. In the present study, the expression levels of several transcripts involved in cholesterol metabolism, such as *cPLA2α*, *APOB*, *ABCA7*, *SOAT1*, *LRP2*, *LRP1*, *APOC3*, and *LIPG*, were observed to be specially up-regulated in the E41 group; this finding indicated that the function of DGs might be related to the lysosomal exocytosis of lipids. These suggested functions are similar to those of the mandibular gland and the cloacal musk gland reported for Alligator mississipiensis, which possess a smooth, oily substance with a powerful odor [4]. These two sets of integumented glands have also been observed in *Alligator sinensis* and are thought to be associated with sexual activity and epigamic in the breeding population. Among *Alligator sinensis*, DGs are proposed to be degenerate organs; this hypothesis is based only on the evidence of an absence of a glandular duct opening on the skin surface, which may be absent or blocked by the proliferation of epithelial cells in late embryonic development [15]. However, whether DGs have functions similar to those of integumented glands remains to be further investigated among adult individuals.

A large number of studies have shown that members of the PITX family—as important transcription factors regulating growth and development—are involved in multiorgan development during vertebrate embryo development. For instance, PITX1 has an important transcriptional-regulation role in the expression of a corticotroph-specific gene which is responsible for bicoid-related homeoproteins [39]. PITX1 has been identified as a novel gene potentially playing a role in the hemangioblast-derived hemogenic lineage in early chick embryos [40]. It has been reported that PITXs are sufficient and necessary for the induction of ectopic cement gland formation; this hypothesis is supported by the evidence of PITX1 and PITX2 knockdown, which leads to the abnormal differentiation of this anterior-most ectodermal organ of Xenopus embryos [41]. PITX1 promotes cement gland differentiation through its function as a transcriptional activator, which inhibits BMP signaling [42]. It has been accepted that PITX1 has the potential to inhibit telomerase activity, leading to the suppression of cell viability and the induction of apoptosis [43]. PITX suppresses TERT transcription through the direct binding to the TERT promoter, which ultimately regulates telomerase activity [44]. Recently, it has been proposed that circ-PITX1 exerts itself as a competing endogenous RNA by sponging miR-379-5p to elevate the expression of MAP3K2 and rescue the cell proliferation and apoptosis within the glioblastoma; this is regarded as the most common type of primary malignant tumor to occur in the human central nervous systems [45]. In the mouse abdominal wall, PITX2 acts as an inhibitor of protein transport and cell apoptosis, contributing to the open-body wall phenotype [46]. In terms of cell cycle regulation, PITX2 up-regulation induces a rapid reduction in cell proliferation, which is associated with an accumulation of neural stem cells in the G1 phase, via promoting p21 expression and inducing a cell cycle exit among neural progenitors [47]. In the current research, in accordance with the transcriptome sequencing data, the IHC results showed that the moderately abundant PITX2-positive signals mainly appeared in the SCs collected from the E36 samples. However, sporadically weak PITX2 was observed to be expressed within the BCs and SCs collected from the E41 and NH1 stage samples. The corresponding dynamic expression patterns of PITX2 were opposite to those of cell apoptosis indication via a TUNEL reaction within the secretory and degenerative layers when they developed to the NH1 stage; this finding further suggested that PITX2 maintains the antiapoptotic defense in the DGs of crocodilian species.

## 5. Conclusions

Firstly, the cell proliferation and apoptosis examinations were designed to provide the molecular evidence directly supporting for the DG serving as a multicellular exocrine glands via holocrine secretion. The corresponding works not only provided a basis information of glandular secretory mode of DG but also confirmed the DG was indeed a functional epidermal gland rather than a degenerate or inactive organ/tissue. Furthermore, using the RNA-seq, comprehensive transcriptional changes in a temporal manner surrounding the incubation period were conducted to identify the candidate genes for further elucidate its possible function in the transition from amniotic fluid environment to terrestrial environment around the timepoint of hatching. Finally, as the identified DEGs in the RNA-seq, the Pitx2 is also known as a key transcription factor functioning in epidermal keratinocyte differentiation. In subsequent works involving phylogenetic and immunohistochemical analysis, it would be helpful to further elucidate the transcriptional regulatory mechanism of DEGs obtained by the previous RNA-Seq. In conclusion, the present results are of considerable importance for enriching the understanding of the intrinsic relationship between the skin derivatives and lifestyles of *Alligator sinesis*.

## Figures and Tables

**Table 1:** The list of DEGs involved in the cholesterol metabolism, melanogenesis, and cell apoptosis pathways.

Pathways	Gene ID	Gene Names	Log_2_ Fold Change		Gene Description
			E36 vs. E41	E41 vs. NH1	
Cholesterol metabolism (asn04979)	102386454	cPLA2α	−2.86	1.36	Cytosolic phospholipase A2-epsilon
	102383976	APOB	−4.23	5.42	Apolipoprotein B
	102387412	ABCA7	−1.72	-	ATP-binding cassette subfamily A member 7
	102373020	SOAT1	−1.43	-	Sterol O-acyltransferase 1 transcript variant X1
	102386183	LRP2	−2.37	3.81	Low density lipoprotein receptor-related protein 2
	102370878	LRP1	−1.10	-	Low density lipoprotein receptor-related protein 1
	102370489	APOC3	−5.75	9.29	Apolipoprotein C-3
	102378969	LIPG	−1.12	1.73	Lipase endothelial
	102382151	PCSK9	-	2.39	Proprotein convertase subtilisin/kexin type 9
	102370247	APOA-1	-	3.27	Apolipoprotein A-I
	102373321	TSPO	-	2.66	Translocator protein 2
Melanogenesis (asn04916)	102375492	Kitl	−1.79	1.83	KIT ligand
	novel.2464	HMG	−2.26	-	High mobility group box
	102373200	WNT5a	−1.30	1.06	Wingless-type MMTV integration site family member 5A
	102379576	FZD7	−1.48	-	Frizzled class receptor 7
	102371525	FZD10	−1.40	2.74	Frizzled class receptor 10
	102374249	ADCY3	−1.26	-	Adenylate cyclase 3 transcript variant X1
	102371353	PLCB3	−1.12	-	Phospholipase C beta 3 (phosphatidylinositol-specific)
	102371988	FZD1	−1.01	1.07	Frizzled class receptor 1
	102378044	DCT	-	4.95	Dopachrome tautomerase transcript variant X2
	102385049	TYR	-	3.10	Tyrosinase
	102380912	MC1R	-	3.36	Melanocortin 1 receptor
	102367949	TYRP1	-	1.96	Tyrosinase-related protein 1
	102376031	WNT4	-	2.05	Wingless-type MMTV integration site family member 4
	102371518	WNT16	-	6.43	Wingless-type MMTV integration site family member 16
	102377243	WNT6	-	1.25	Wingless-type MMTV integration site family member 6
Cell Apoptosis (asn04210)	102387989	MCP1	3.68	-	Mast cell protease 1A-like
	102376560	Tubulin α-3	4.13	-	Tubulin alpha-3 chain-like transcript variant X2
	102377814	CRLM2	2.57	-	Cytokine receptor common subunit beta-like
	102377569	CSF2RB	2.56	-	Colony stimulating factor 2 receptor beta low-affinity
	102380435	BIRC5	1.189	-	Baculoviral IAP repeat containing 5
	102378651	GPX2	−1.55	1.95	Glutathione peroxidase 2
	102384495	APOC1	-	1.15	Apolipoprotein C-I
	102386964	CTSW	-	−2.12	Cathepsin W-like
	102374632	Tubulin α-8	-	−1.78	Tubulin alpha 8
	102375471	FAS	-	−2.08	Fas cell surface death receptor
	102379727	ITPR2	-	−1.29	Inositol 1 4 5-trisphosphate receptor type 2
	102381984	Bcl2	-	−1.37	BCL2-like 11 (apoptosis facilitator)

The fold change of E36 vs. E41 = RPKM in E41/RPKM in E36; The fold change of E41 vs. NH1 = RPKM in NH1/RPKM in E41.

## Data Availability

The data presented in this study are available on request from the corresponding author (E-mail address: wuxb@ahnu.edu.cn, X. Wu).

## Supplementary Materials

The following supporting information can be downloaded at: https://www.mdpi.com/article/10.3390/life12111787/s1, Figure S1: The purity and integrity detection of isolated RNA using electrophoresis (A) and Agilent assay (B); Table S1: Parameters of RNA Quality control; Table S2: Summary of data output quality; Table S3: A summary of the sequencing reads alignment; Table S4. Details of primer sequences, product sizes and accession numbers of genes using for RT-PCR; Table S5: Information of other organisms PITX2 sequences collected from NCBI; Figure S2: The timepoint pattern changes in morphological characteristics and proportion of melanin observed in the limbs scales, tail scales, and back scales of embryonic and new-hatched *A. sinesis* collected from different developmental stages including E31, E36, E41, E45, E54, and NH1.
