# Supplementary material for: RNA-Sequencing Analysis of Gene-Expression Profiles in the Dorsal Gland of Alligator sinensis at Different Time Points of Embryonic and Neonatal Development
Source: Life (Basel). 2022 Nov 4;12(11):1787. doi: 10.3390/life12111787 (PMC9697384; doi:10.3390/life12111787)
Supplement: Supplementary file 1 [file life-12-01787-s001.zip › life-2005279-supplementary.pdf]

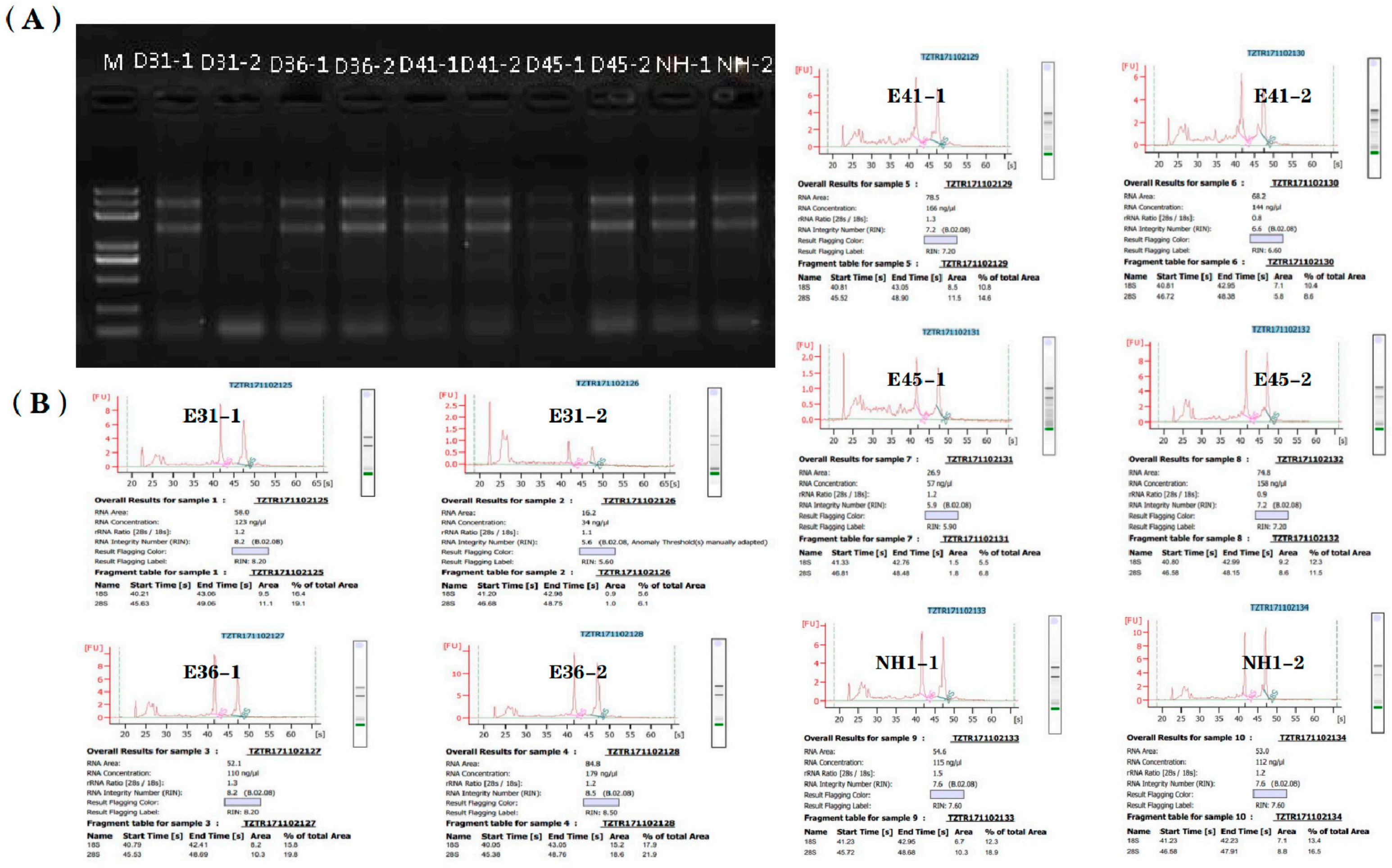

Figure S1. The purity and integrity detection of isolated RNA using electrophoresis (A) and Agilent assay (B).

Table S1. Parameters of RNA Quality control

Sample name	RNA concentration (ng/μl)	Total RNA content (ug)	OD260/280	OD260/230	28S/18S	RIN value
E31-1	123	2.706	1.789	1.214	1.2	8.2
E31-2	32	0.704	2.286	0.8	1.1	5.6
E36-1	220	4.840	1.969	1.703	1.3	8.2
E36-2	486	15.552	1.96	1.96	1.2	8.5
E41-1	476	10.472	1.904	1.951	1.3	7.2
E41-2	278	6.116	1.904	1.479	0.8	6.6
E45-1	57	1.254	1.752	1.129	1.2	5.9
E45-2	158	3.476	1.804	1.137	0.9	7.2
NH1-1	230	5.060	1.889	1.526	1.5	7.6
NH1-2	336	7.392	2.012	1.899	1.2	7.6

Table S2. Summary of data output quality

Sample name	Raw reads	Clean reads	Clean bases	Error rate(%)	Q20(%)	Q30(%)	GC content(%)
E31-1	33467342	32901422	9.87G	0.02	97.67	96.29	52.94
E31-2	38661829	37977196	11.39 G	0.03	97.16	92.40	53.37
E36-1	46596735	45800617	13.74 G	0.03	97.08	95.45	53.53
E36-2	33152584	32634069	9.79 G	0.03	97.61	93.65	52.27
E41-1	40598536	39680358	11.9 G	0.03	96.69	94.90	54.67
E41-2	41948456	41119740	12.34 G	0.03	97.02	95.37	53.06
E45-1	24708654	24260615	7.28 G	0.03	96.44	95.02	53.18
E45-2	36347232	35886664	10.77 G	0.03	97.47	93.32	52.88
NH1-1	39642619	39003489	11.7 G	0.02	98.22	94.88	53.51
NH1-2	26837040	26480528	7.94 G	0.03	96.52	94.88	54.41

Table S3. A summary of the sequencing reads alignment

Sample	Total_Reads	Total_Map	Unique_Map	Multi_Map	Read1_Map	Read2_Map	Positive_Map	Negative_Map	Splice_Map	Unsplice_Map	Proper_Map
E31_1	65802844	57211013(86.94%)	55695532(84.64%)	1515481(2.3%)	27923564(42.44%)	27771968(42.2%)	27906196(42.41%)	27789336(42.23%)	24474640(37.19%)	31220892(47.45%)	52188232(79.31%)
E31_2	75954392	65310956(85.99%)	63377837(83.44%)	1933119(2.55%)	31819769(41.89%)	31558068(41.55%)	31742276(41.79%)	31635561(41.65%)	30858924(40.63%)	32518913(42.81%)	59503200(78.34%)
E36_1	91601234	78666783(85.88%)	76273667(83.27%)	2393116(2.61%)	38257393(41.77%)	38016274(41.5%)	38287292(41.8%)	37986375(41.47%)	38968753(42.54%)	37304914(40.73%)	71175026(77.7%)
E36_2	65268138	57289288(87.78%)	55643794(85.25%)	1645494(2.52%)	27866875(42.7%)	27776919(42.56%)	27896779(42.74%)	27747015(42.51%)	26380657(40.42%)	29263137(44.84%)	52059074(79.76%)
E41_1	79360716	67075529(84.52%)	65238352(82.2%)	1837177(2.31%)	32807704(41.34%)	32430648(40.86%)	32640652(41.13%)	32597700(41.08%)	27712611(34.92%)	37525741(47.29%)	60657408(76.43%)
E41_2	82239480	71137627(86.5%)	69134727(84.07%)	2002900(2.44%)	34697037(42.19%)	34437690(41.87%)	34626668(42.1%)	34508059(41.96%)	29689391(36.1%)	39445336(47.96%)	64727996(78.71%)
E45_1	48521230	42062777(86.69%)	40533236(83.54%)	1529541(3.15%)	20336663(41.91%)	20196573(41.62%)	20269759(41.78%)	20263477(41.76%)	13026089(26.85%)	27507147(56.69%)	37843956(77.99%)
E45_2	71773328	62161767(86.61%)	59935538(83.51%)	2226229(3.1%)	30083993(41.92%)	29851545(41.59%)	30252669(42.15%)	29682869(41.36%)	28411564(39.59%)	31523974(43.92%)	55166278(76.86%)
NH1_1	78006978	67728172(86.82%)	65471874(83.93%)	2256298(2.89%)	32802190(42.05%)	32669684(41.88%)	33027380(42.34%)	32444494(41.59%)	30105891(38.59%)	35365983(45.34%)	61032662(78.24%)
NH1_2	52961056	45286727(85.51%)	43565283(82.26%)	1721444(3.25%)	21875673(41.31%)	21689610(40.95%)	22114865(41.76%)	21450418(40.5%)	23218642(43.84%)	20346641(38.42%)	40183964(75.87%)

Table S4. Details of primer sequences, product sizes and accession numbers of genes using for RT-PCR

Genes	Accession number	Primer sequences (5′-3′)		Production size (bp)
		Forward	Reverse	
KIT ligand	XM_025192289.1	ACAGTTCCATGTTCGCTTCA	TCCAAAAATGCCACGCAAGG	244
FZD7	XM_006039408.3	CAAGACCCTACAGTCCTGGC	GAAGGGCACTTAGTCCGCAA	197
WNT5A	XM_025197125.1	AGGCCCAAAGATTTACCCCG	ACTTACAGGCCACATCAGCC	214
TYR	XM_006030556.3	GGTGGGTCGAGCATATGGAG	ATAGGCCCTTCATTGGTGGC	289
MC1R	XM_006027861.2	CCCCTCATCTATGCCTTCCG	CTGTCATGCACCCCTCAGTC	157
BIRC5	XM_006030455.3	GGCGGAGGCAGATCTACAAT	AATGTAGCGACGCATGTCCT	463
TUbA3	XM_006032942.3	GACCTTGAGCCAACAGTGGT	CCAGTGCCTCCTCCAAAACT	233
FAS	XM_014522432.2	TCACCGTGCAACAATGGAGT	TCCTGTGCCGAAAGCAGTAG	340
BCL-2	XM_025194199.1	ACAATGGAGGCTGGTGTTGG	AGCTTTGGGCATTTTGTGCTT	431
CPLA2α	XM_014524902.2	GTATCGGCGGAGTCAAGAGG	ATCTCGGGGAACAACCACAC	508
PLD4	XM_006036353.3	GCTCCCAAGATGGGTGAGAG	TCCAGGCTTGGTCTAAGGGT	394
CYP2J2	XM_019479193.1	GTACACACGGGGACAGAGTG	GAGGGAGGCTAGCTTTGACC	290
Alox5	XM_006014831	AGTATTCAAGGCGCAGGGAC	ATGTCACGGGGCAAATCACT	382
GPX2	XM_006035816.3	CGGGATTACACCCAGCTCAA	AAGTTCCAGGTCACGTCTGC	329
TUbA3	XM_025194939.1	CATGCTGGCGGTACGAAATG	GGGCTGAATCCCATGTTCCA	190
RPL8	XM_006015645	GGTGTGGCTATGAATCCTGT	ACGACGAGCAGCAATAAGAC	253
PITX-2	MK992783	CGGCAGAGGACTCACTTCAC	CATCGTAGGGCTGCATGAGA	247
β-actin	KC286488.1	GTACCACCATGTACCCAGGC	TCGGTGTGACGTGGTAACAG	250

KITLG: Kit Ligand, *FZD7:* Frizzled Class Receptor 7, *WNT5A:* Wingless-Type Integration Site Member 5A, *TYR:* Tyrosinase, *MC1R:* Melanocortin-1 Receptor, *BIRC5:* baculoviral IAP repeat containing 5, *TUBA3:* tubulin alpha-3 chain, *FAS:* Fas cell surface death receptor, *CPLA2α:* Cytosolic Phospholipase-A2 Epsilon, *PLD4:* Phospholipase D Member 4, *CYP2J2:* Cytochrome P450 2J2, *Alox5:* Arachidonate 5-Lipoxygenase. *GPX2:* Glutathione Peroxidase-2, *TUBA8:* tubulin alpha-8 chain, *RPL8*: Ribosomal protein L8.

Table S5. Information of other organisms PITX2 sequences collected from NCBI

Species name	Accession number
Danio rerio	AAD34390.1
Podarcis muralis	XP_028599367.1
Xenopus laevis	AAC29426.1
Oreochromis niloticus	XP_003443491.1
Anas platyrhynchos	XP_027312253.1
Macaca mulatta	XP_001091288.1
Oryzias latipes	XP_023814716.1
Homo sapiens	NP_000316.2
Mus musculus	NP_001035967.1
Gallus gallus	AAC27322.1
Cricetulus griseus	XP_027251219.1
Pelodiscus sinensis	XP_025038495.1
Alligator mississippiensis	XP_014450876.1（PREDICTED）

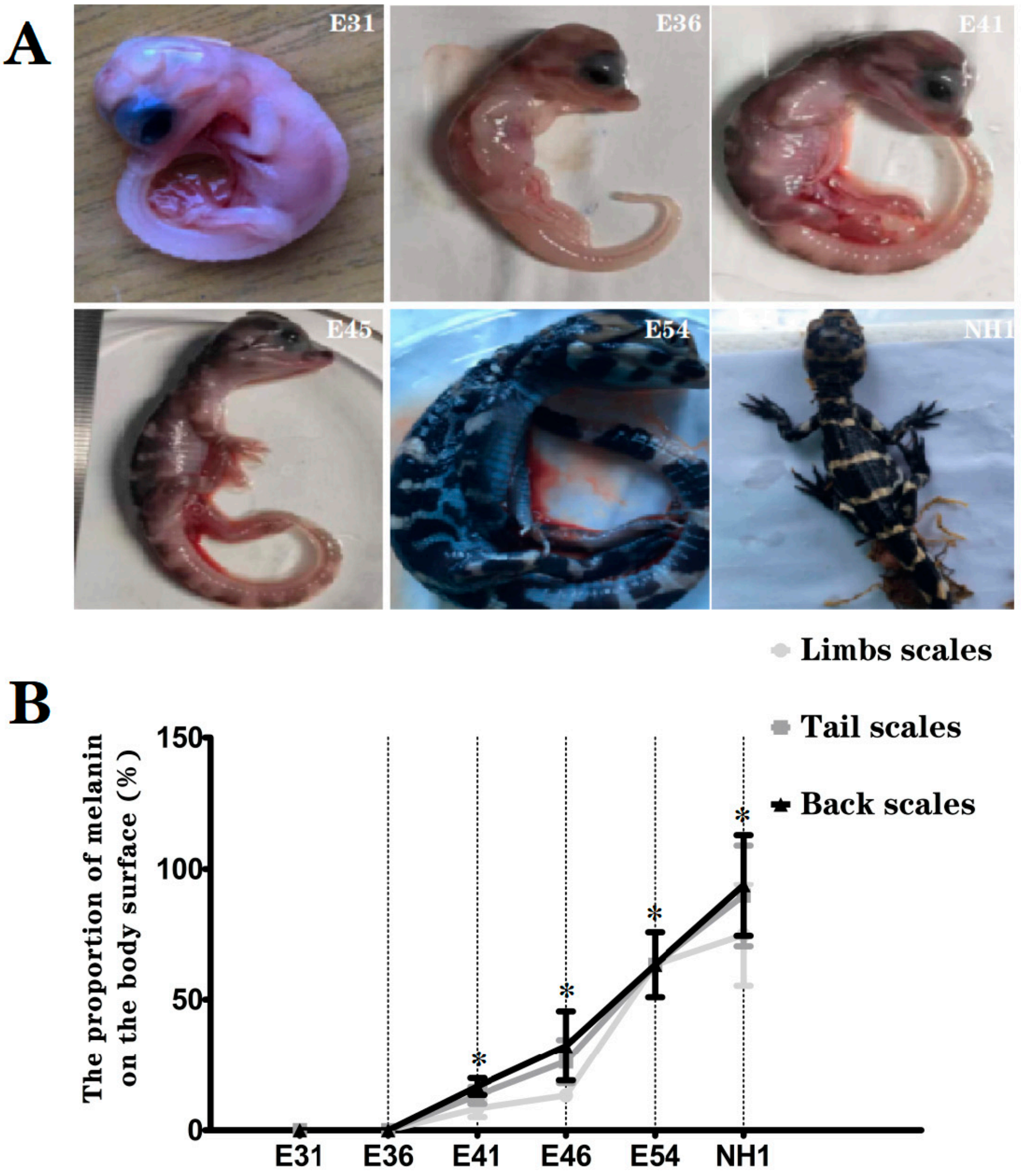

Figure S2. The time-point changes pattern in morphological characteristics and proportion of melanin observed in the limbs scales, tail scales, and back scales of embryonic and new-hatched *A. sinesis* collected from different developmental stages including E31, E36, E41, E45, E54 and NH1.
